# P10s-PADRE vaccine combined with neoadjuvant chemotherapy in ER-positive breast cancer patients induces humoral and cellular immune responses

**DOI:** 10.18632/oncotarget.28083

**Published:** 2021-10-26

**Authors:** Issam Makhoul, Saddam Mohammed Ibrahim, Muhammad Abu-Rmaileh, Fariba Jousheghany, Eric R. Siegel, Lora J. Rogers, John J. Lee, Sergio Pina-Oviedo, Ginell R. Post, J. Thaddeus Beck, Thomas Kieber-Emmons, Behjatolah Monzavi-Karbassi

**Affiliations:** ^1^Department of Medicine, University of Arkansas for Medical Sciences, Little Rock, AR 72205, USA; ^2^Department of Pathology, University of Arkansas for Medical Sciences, Little Rock, AR 72205, USA; ^3^Department of Biostatistics, University of Arkansas for Medical Sciences, Little Rock, AR 72205, USA; ^4^Department of Internal Medicine, University of Texas Southwestern, Dallas, TX 75390, USA; ^5^Highland Oncology Group, Fayetteville, AR 72703, USA; ^6^Winthrop P. Rockefeller Cancer Institute, University of Arkansas for Medical Sciences, Little Rock, AR 72205, USA; ^7^UnivLyon, Université Claude Bernard Lyon 1, Villeurbanne 69100, France

**Keywords:** cancer vaccine, peptide mimotopes, combination therapy, breast cancer

## Abstract

Breast cancer patients diagnosed with HR+/HER2– tumors face a persistent risk of distant recurrence long after completion of their treatment. Strategies to induce anti-tumor immune responses could complement standard-of-care therapies for these patients. The current study was performed to examine the feasibility, safety and immunogenicity of adding P10s-PADRE to standard-of-care chemotherapy in HR+/HER2− early-stage breast cancer patients. Twenty-five subjects were treated in a single-arm Phase Ib clinical trial. Five different immunization schedules were considered to evaluate the feasibility of eliciting an immune response. The primary immunogenicity endpoint was antibody titer. The expression of several activation markers on natural killer (NK) cells and serum concentrations of Th1/Th2 cytokines were also examined. The percentage of tumor-infiltrating lymphocytes (TILs) was determined. Antibody response was superior in schedule C where 3 weekly immunizations preceded the first dose of chemotherapy. A significant change in CD16, NKp46 and CD94 expression levels on NK cells and a rise in serum content of IFN-γ was observed after treatment. Schedule C showed an increase in TILs in residual lesions. The combination therapy is safe and immunogenic with treatment schedule C being immunologically promising. Randomized trials focused on long-term survival outcomes are needed to evaluate clinical benefits.

## INTRODUCTION

HR+/HER2− breast cancer remains the most common form of breast cancer in the United States [1]. The positive prognostic effect of ER+ status is limited to 5 years after diagnosis, as the prognosis of HR+ patients becomes worse than that of triple-negative or HER2+ patients among those alive after 5 years since diagnosis [2–4]. Apparently, the current paradigm of endocrine therapy with or without chemotherapy has not been effective for a considerable number of these patients [5, 6]. Neoadjuvant chemotherapy (NAC) has become a standard practice for treatment of high-risk localized breast cancer, and pathological complete response (pCR) to NAC has been a trusted surrogate to assess long-term survival benefits. However, for HR+ breast cancer patients, compared to patients with triple-negative or HER2+ tumors, the benefit of NAC in terms of pCR is limited, as pCR to neoadjuvant treatment is observed less frequently in HR+ patients [7, 8]. Residual tumors at the time of surgery in patients treated with NAC are immune-suppressed as evidenced by having a lower abundance of TILs, decreased fraction of immune effectors and increased density of immune-suppressive cells compared to baseline levels [9]. HR+ tumors are notorious for their low immunogenicity, low percentage of TILs, and lack of correlation of TIL levels with favorable clinical outcomes [8]. However, in a study with about 30 years of follow-up, in HR+/HER2− premenopausal breast cancer patients, high infiltration of TILs was a favorable prognostic factor regarding disease recurrence or breast cancer-specific death [10]. In another study, the increase in stromal TILs in residual disease in a patient population, 79% of which had HR+ disease, was associated with improved recurrence-free survival [11]. Therefore, a rational combination therapy that enhances the immune-stimulatory properties of NAC, can provide long-term survival benefits for this patient population. Development of new strategies aimed at active induction of anti-tumor immune responses and improving immunogenicity of such immunologically cold tumors could increase the effectiveness of standard approaches and improve long-term survival benefits.

We have developed carbohydrate-mimetic peptides (CMPs) as a means to augment immune responses to tumor-associated carbohydrate antigens [12, 13]. We have brought one CMP, called P10s-PADRE, to the clinic [14, 15]. We performed a Phase I clinical trial with the P10s-PADRE vaccine in Stage IV breast cancer patients. Immunization induced anti-peptide and anti-glycan antibody responses whereby the induced antibodies were cytotoxic in a Caspase 3-dependent manner [15]. The immunization of breast cancer patients with P10s-PADRE proved feasible, tolerable and immunologically efficacious. After more than 8 years of follow-up, 4 out of 6 vaccinated subjects from the Phase I clinical trial are still alive, with 3 of them in stable condition and 1 subject still in remission. Moreover, we observed that P10s-PADRE-induced immune serum triggered chemosensitivity to paclitaxel in the HR+ZR-75-1 cell line [15], which suggests that combining the P10s-PADRE vaccine with chemotherapy regimens that include taxanes may prove beneficial in treating breast cancer patients.

In our preclinical studies, we observed that natural killer (NK) cells were involved in P10s-mediated tumor eradication, and that the data generated in the Phase I clinical trial of P10s-PADRE indicate activation of NK cells, as shown by increased expression of the NKp46 (NCR1, natural cytotoxicity triggering receptor 1) marker on circulating NK cells [16]. NK cells are CD3^–^/CD56^+^ (NCAM1) lymphocytes that constitute about 10% of peripheral blood lymphocytes, and are considered a component of the innate immune system that can control tumors and microbial infections through their ability to produce cytokines like IFN-γ and TNF-α as well as their cytolytic activity [17]. The potential of increased NK-mediated immune response has been associated with rejection of lenalidomide-resistant multiple myeloma tumor [18] and improved clinical response in neuroblastoma patients [19]. Additionally for a vaccine with antibody-dependent anti-tumor mechanisms of action, CD16 receptor is considered a potent activator of NK cells and the main mediator of antibody-dependent cell-mediated cytotoxicity (ADCC).

The data derived from a Phase Ib clinical trial in HR+/HER2− breast cancer patients shown here indicate that combining P10s-PADRE with NAC is feasible and immunogenic.

## RESULTS

### Subject characteristics, adverse events and feasibility

Characteristics of subjects enrolled and vaccinated are summarized in Table 1. Adverse events (AEs) were graded and evaluated at each visit throughout the duration of the study (about 16 months after application of the first dose of the vaccine or chemotherapy). A total of 626 AEs were recorded, with 42 AEs attributed as definite, probable, or possible relationship to the vaccine (Table 2). Twenty-seven AEs were described as grades 1 and 2 injection-site reactions that included redness, rash, induration, itching, burning, warmth, and sometimes pain. Only two AEs were described as grade 3. They included one severe abdominal distention and one severe fatigue. No grade ≥4 AEs were observed. All patients completed their treatment schedule. The data suggest that adding P10s-PADRE vaccine to NAC was feasible and well-tolerated, with injection-site irritation as the only repetitive AE.

**Table 1:** Characteristics of subjects accrued

Subject study number	Race	Age	PS^*^	Tumor stage, Tumor grade	Tumor type^+^	Tumor size at baseline (cm)^#^	Tumor size after treatment (cm)^$^	Clinical LN status at baseline^#^	Pathologic LN status after treatment^$^
**A1**	W	58	0	IIB, 1	A	4.2	0.2	N1	N1
**A2**	W	64	0	IIIB, 2	A	3.2	0.5	N0	N0
**A3**	W	40	0	IIB, 2	B	2.6	2	N1	N1
**A4**	W	74	1	IIA, 2	B	2.5	4.4	N0	N0
**A5**	W	59	0	IIB, 2	B	2	0.2	N1	N1
**B1**	W	55	0	IIA, 3	B	3.7	0	N0	N0
**B2**	W	49	0	IIIB, 3	A	4	0.5	N1	N0
**B3**	AA	55	0	IIA, 2	A	3.3	3.7	N0	N0
**B4**	W	67	0	IIB, 2	A	2.8	1.5	N1	N0
**B5**	AA	39	0	IIIA, 2	A	6.5	0.1	N1	N1
**C1**	W	63	0	IIIB, 1	A	7	3.5	N1	N1
**C2**	W	60	0	IIIB, 2	B	3.7	3.5	N0	N1
**C3**	W	40	0	IIB, 3	A	2.7	1.1	N1	N1
**C4**	W	71	0	IIB, 2	A	7	7.5	N0	N1
**C5**	W	54	0	IIA, 3	B	3.7	0.3	N0	N0
**D1**	W	54	0	IIB, 2	A	4.9	2.5	N0	N0
**D2**	W	70	0	IIA, 3	B	1.3	0	N0	N0
**D3**	W	45	0	IIB, 2	A	4.6	4	N1	N1
**D4**	W	70	0	II, 3	B	1	0.4	N1	N1
**D5**	W	35	1	III, 2	B	7	0.5	N1	N0
**E1**	W	45	1	IIB, 2	A	1.4	3	N1	N0
**E2**	W	67	0	IIA, 2	B	2.4	1.7	N0	N1
**E3**	W	45	0	IIIB, 3	B	2.5	2.4	N0	N0
**E4**	W	32	0	IIA, 3	B	2.7	0.2	N0	N0
**E5**	W	28	0	IIB, 3	B	0.8	0	N1	N1

^*^Performance status (ECOG); ^+^Luminal A vs. Luminal B; ^#^Clinical examination; ^$^Pathological examination.

**Table 2:** Number of AEs (number of patients, pts) by grade, where relationship to vaccine is definite, probable, or possible

Body system	Adverse event	Severity scale grade						All AEs
		1: Mild		2: Moderate		3: Severe		
		AEs	(pts)	AEs	(pts)	AEs	(pts)	
Gastrointestinal disorders	Abdominal distension	–	–	1	(1)	1	(1)	2
General disorders and administrative site conditions	Fatigue	1	(1)	2	(2)	1	(1)	4
	Injection site reaction	23	(23)	4	(3)	–	–	27
	intermittent fatigue	1	(1)	2	(2)	–	–	3
Musculoskeletal and connective tissue disorders	Musculoskeletal and connective tissue disorders - other, sharp pains in random spots	–	–	1	(1)	–	–	1
Nervous system disorders	Headache	–	–	1	(1)	–	–	1
	Intermittent headaches	–	–	1	(1)	–	–	1
Reproductive system and breast disorders	Intermittent left breast pain	1	(1)	–	–	–	–	1
	Reproductive system and breast disorders - other, burning sensation left breast	1	(1)	–	–	–	–	1
Vascular disorders	Vascular disorders – other, night sweats	1	(1)	–	–	–	–	1
All AEs		28	(28)	12	(11)	2	(2)	42

### P10s-PADRE immunization in combination with chemotherapy induced antibody response

**Figure 1:**
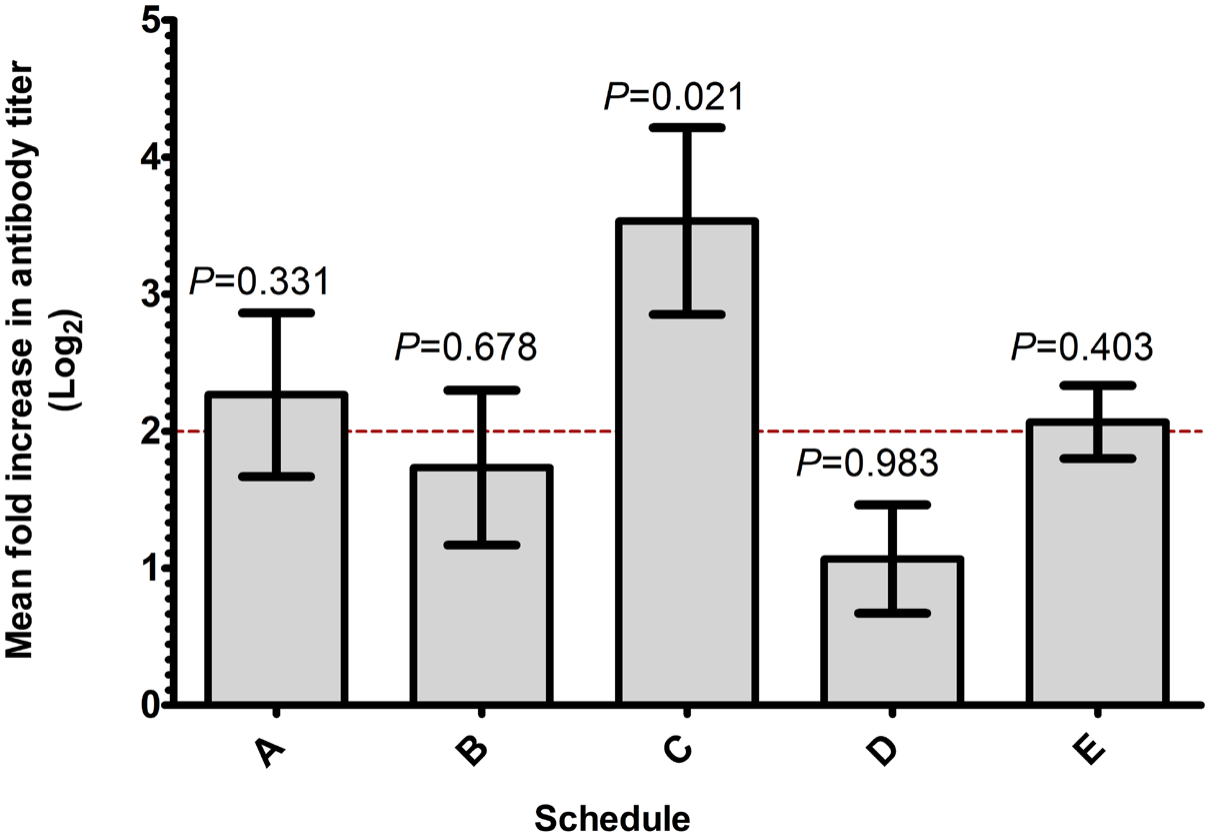
Schedule C produces more consistent and superior antibody titer. Mean fold increase in antibody titers for treatment schedules A through E is shown. Fold increase in anti-P10s peptide titer of 3 consecutive weeks, early during the course of therapy, for all subjects in each schedule was log2-transformed and analyzed using 1-sided *t*-test comparing the mean fold increase with 4 fold.

In the current clinical trial, we used anti-peptide antibody response as a marker to choose the best schedule for combination therapy. Blood specimens were collected at various weeks during each schedule’s period, and fold change in anti-peptide antibody titer was measured (Table 3). We have reported that serum antibodies become detectable as early as week 4 of the study, peak at week 7 and stay stable up to at least the 24th week [13]. In the current study, surgery was conducted between weeks 26 and 33, depending on the combination schedule (Supplementary Table 2). As a criterion for successful immune activation in the designed protocol, we defined a response to consist of at least a 4-fold increase in antibody titer after immunization that repeats in two separate weeks by week 13 to 16, depending on the schedule. Any combination schedule with at least 4 out of 5 subjects responding was considered as an acceptable schedule for future clinical trials. According to the data summarized in Table 3, both schedules C and E qualified as acceptable, with each of them having 4 subjects responding to the immunization by week 16. Further comparisons of the magnitude of antibody response (Figure 1) indicate that mean fold increase in antibody titer of schedule C (48-fold increase) is significantly higher than the 4-fold limit (*P* = 0.021). The data suggest that subjects enrolled in schedule C generated a more consistent and robust antibody response, therefore schedule C appears as the schedule of choice for future combination therapy. However, continued analysis of blood samples collected suggests that, in patients enrolled on the other schedules, serum antibodies may start to appear later, close to surgery or even later (i.e., please see Table 3, first and second subjects of schedule B).

**Table 3:** Fold increase in anti-peptide IgG titer in subjects enrolled in 5 schedules

Group	W-4	W-7	W-10	W-13	W-22	W-46	W-70
**A**	4	32	32	16	16	16	8
**A**	1	4	4	2	2	2	2
**A**	NA	NA	64	32	16	16	32
**A**	1	1	1	N/A	N/A	N/A	N/A
**A**	1	1	1	1	1	1	1
	**W-7**	**W-10**	**W-13**	**W-16**	**W-22**	**W-46**	**W-70**
**B**	1	1	1	1	1	8	8
**B**	1	1	1	1	4	8	8
**B**	4	16	16	64	32	16	N/A
**B**	1	1	2	2	2	1	2
**B**	16	32	32	32	8	4	4
	**W-7**	**W-10**	**W-13**	**W-16**	**W-25**	**W-49**	**W-73**
**C**	8	32	32	4	4	2	2
**C**	4	4	4	32	32	32	N/A
**C**	8	16	32	64	64	64	N/A
**C**	1	1	1	1	1	8	4
**C**	64	256	256	256	64	16	32
	**W-5**	**W-8**	**W-11**	**W-14**	**W-23**	**W-47**	**W-71**
**D**	1	4	4	8	N/A	8	N/A
**D**	1	1	1	1	1	2	2
**D**	8	16	16	8	8	16	N/A
**D**	1	1	1	1	2	2	2
**D**	2	1	1	2	1	1	16
	**W-6**	**W-9**	**W-12**	**W-15**	**W-24**	**W-48**	**W-72**
**E**	4	8	32	N/A	N/A	NA	N/A
**E**	2	4	4	8	8	16	8
**E**	2	8	4	N/A	N/A	N/A	N/A
**E**	4	4	4	N/A	N/A	N/A	N/A
**E**	4	2	2	N/A	N/A	N/A	N/A

Antibody titer was determined in pre-immune samples and at various times (weeks, W) after immunization. The fold increase in antibody titer (relative to pre-immune levels) is reported.

### Combination therapy affected the expression levels of NK-cell markers

**Figure 2:**
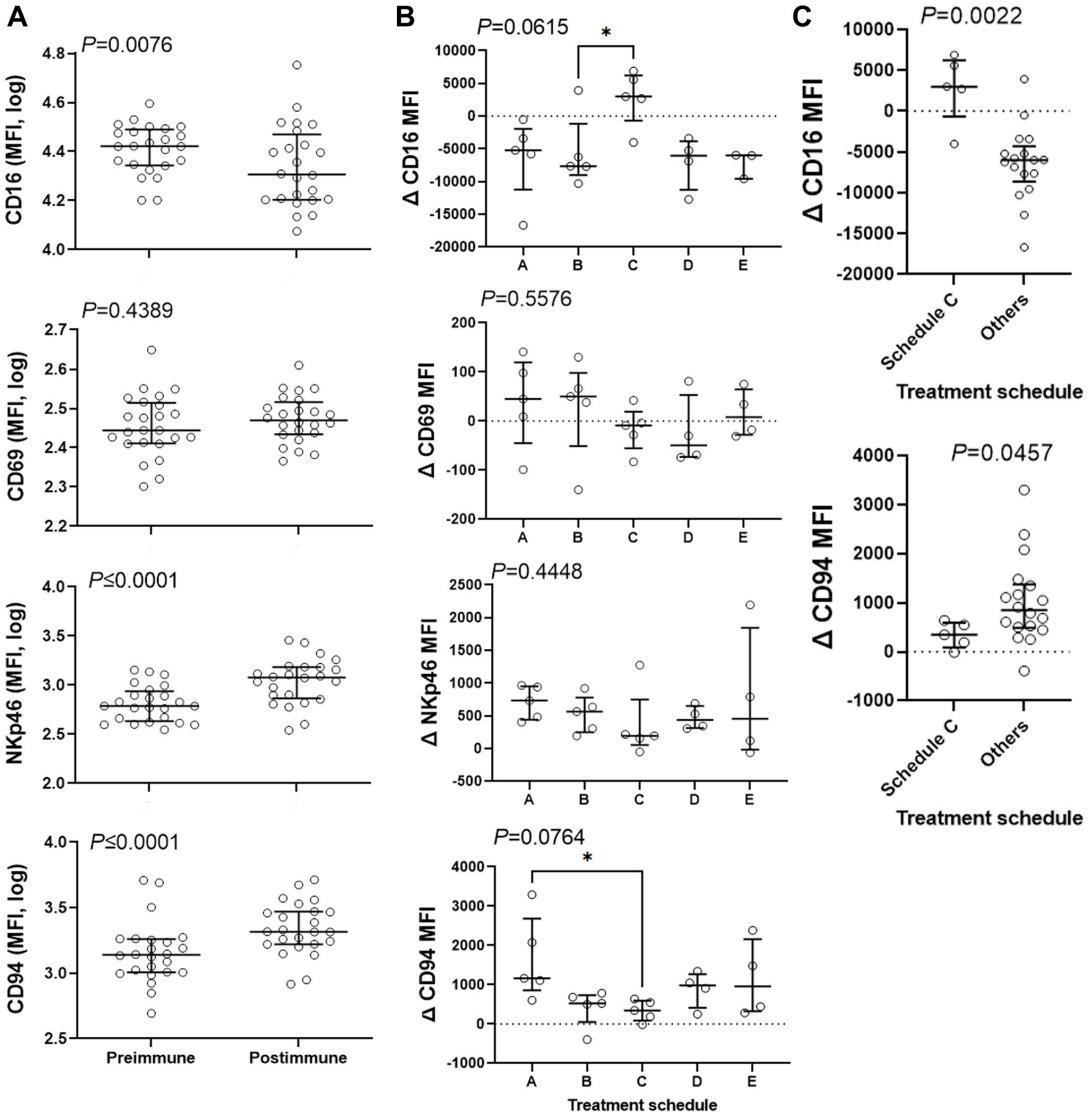
Combination therapy affects expression of NK-cell markers. CD3-negative CD56-positive population was gated and the expression levels of CD16, CD69, NKp46, and CD94 was determined. (**A**) Median Fluorescence Intensity (MFI) for these markers was measured in pre and postimmune samples and was plotted for 23 subjects. Data were analyzed with 2-sided paired Wilcoxon’s signed-rank test. (**B** and **C**) The effect of treatment schedule (the timing of administration of the vaccine relative to chemotherapy) on the therapy-induced change in the expression levels of the above markers was analyzed. Data were analyzed with Kruskal-Wallis (B) or 2-sided Mann-Whitney (C) tests. Kruskal-Wallis test was followed by Dunn’s multiple comparisons test comparing schedule C with other schedules. Bars show median and interquartile range. ^*^, significant at *P* ≤ 0.05.

In the Phase I clinical trial, we explored activation of NK cells by analyzing the expression of several NK-cell markers and observed a statistically significant increase in expression of NKp46 on CD3^–^/CD56^+^ blood lymphocytes [16]. Therefore, we isolated peripheral blood mononuclear cells and used median fluorescence intensity (MFI) to determine the expression of CD16, CD69, NKp46 and CD94 markers in the CD3^–^/CD56^+^ NK population. Change in MFI of the markers in post-immune cells compared to pre-immune cells was plotted (Figure 2A). We observed that the MFI of NKp46 increased from 612 to 1201 (96%, *P* ≤ 0.0001) and that the MFI of CD94 increased from 1377 to 2070 (50%, *P* ≤ 0.0001) in response to treatment when the entire study population was analyzed. Contrary to NKp46 and CD94 expression, CD16 (FcγRIII) expression dropped 23% after immunization (*P* = 0.0076). No significant change in the expression of CD69 (*P* = 0.4389) was observed. Treatment schedule affected the change in the expression of CD16 and CD94 with a tendency towards significance (Figure 2B). Schedule C displays a high and positive MFI for CD16 expression levels and the lowest MFI for CD94 expression levels in response to therapy. Because of the robust antibody production and the elevated expression levels of CD16, schedule C was compared against all other schedules one-by-one and others combined. One-by-one comparisons of schedule C with other schedules suggest a significant increase in CD16 and a significantly different lower levels of CD94 expression compared to schedules B and A, respectively (Figure 2B). Comparison of schedule C with other schedules combined revealed a statistically significant separation between the two regarding CD16 (median of 2999 vs. −6008) and CD94 (median of 346 vs. 849) expression (Figure 2C). The data suggest that schedule C may affect NK cells differently than other schedules. With the most robust antibody response and the upregulation of the Fc receptors, it is expected that schedule C would be the most effective in antibody-mediated targeting and killing of cancer cells.

The expression of CD69 in T (CD3+/CD56-) and NKT (CD3+/CD56+) cells was examined in pre- and post-immune samples. No significant change in MFI of CD69 and no treatment effect was observed (data not shown).

### Combination therapy stimulated production of cytokines

**Figure 3:**
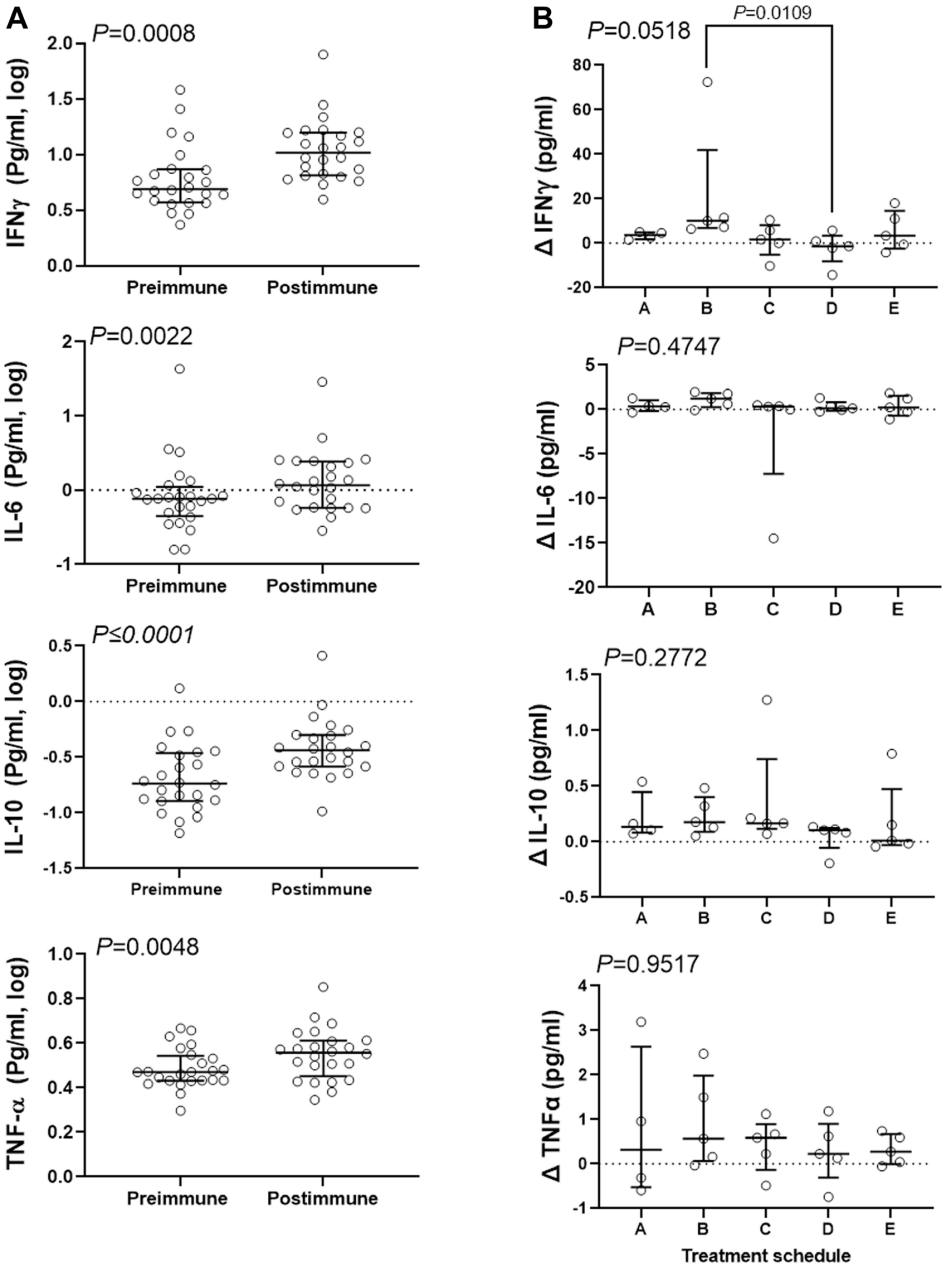
Combination therapy resulted in increase of serum content of several inflammatory cytokines. Concentration of the cytokines was determined in pre- and post-immune serum samples for 24 subjects and analyzed to determine the combination effect in general (**A**), and the effect of treatment schedule in particular (**B**). Data were analyzed with 2-sided paired Wilcoxon’s signed-rank (A) or Kruskal-Wallis (B) tests. Kruskal-Wallis test was followed by Dunn’s multiple comparisons test comparing median change of IFNγ content of schedules B and C with other schedules. Bars show median and interquartile range.

Robust IgG production and activation of NK-cell populations after treatment suggests that immunization with P10s-PADRE may affect cytokine production. The serum concentration of an array of cytokines was measured. The cytokines IL-4 and IL-12 either were not detected or recorded values were below the lower limit of detection. IL-2 and IL-13 contained 5 and 11 undetectable values, respectively, and therefore were removed from further analyses. IL-1β data were also removed from analysis because of a high inter-experimental variability. We detected IL-6, IL-10, IFN-γ, and TNF-α in 24 available serum samples out of 25. Further analysis of the entire cohort indicated significant increases in median concentration of IFN-γ (from 4.9 to 10.4 pg/ml), IL-6 (from 0.76 to 1.17 pg/ml), IL-10 (from 0.18 to 0.36 pg/ml), and TNF-α (from 2.95 to 3.6 pg/ml) in post-immune compared to pre-immune serum samples (Figure 3A). Only IFN-γ increase was affected by treatment schedule, with schedule B showing a median increase of 10.16 pg/ml in post immunization serum compared to schedule D showing a median drop of 1.29 pg/ml in serum content of IFN-γ (Figure 3B). No significant difference between schedule C and other schedules one-by-one or combined was detected.

### T cells are the major component of infiltrating tumor lymphocytes (TILs)

**Figure 4:**
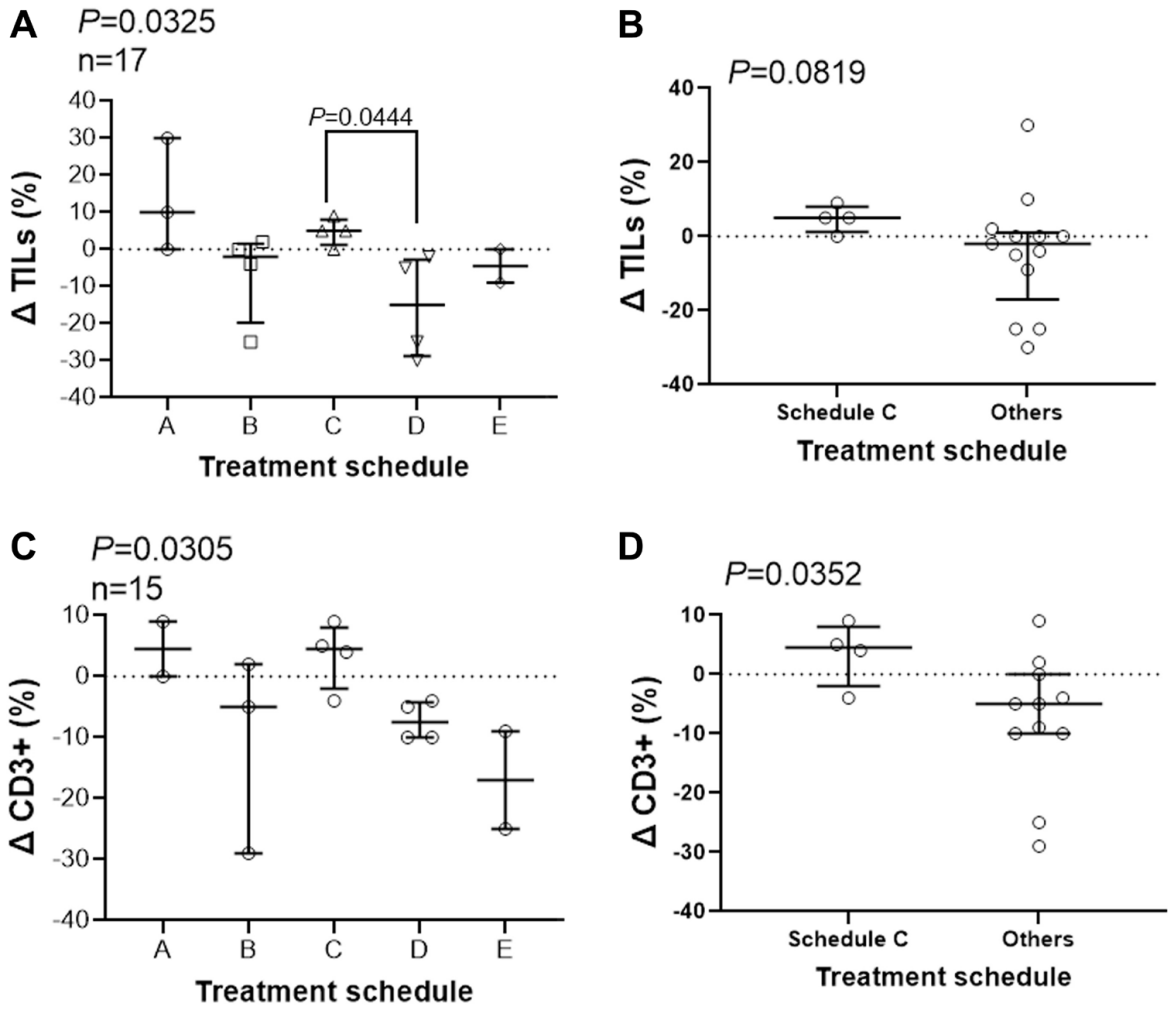
Treatment schedule affect infiltration of immune cells. Slides of core needle biopsies (pre-treatment) and from surgical specimens (post-treatment) were stained with hematoxylin & eosin and an anti-CD-3 antibody and stromal TILs (**A**, **B**) and CD3-positive immune cells (**C**, **D**). Treatment schedules affect the percentage of TILs and CD3+ infiltrates differently using Kruskal-Wallis (A, C) and Mann-Whitney (B, D) test. Dunn’s multiple comparisons test was used post hoc analysis and *P* values for statistically significant differences are shown. Change in percentage of TILs and CD3+ cells for each subject together with median and interquartile range is shown.

Because of the significance of TILs in treatment efficacy [20] and because our data suggests activation of NK cells, we examined the effect of combination therapy on immune response in the tumor microenvironment. We assessed stromal TILs using pre-treatment core biopsies and post-treatment surgical biopsies. Only 17 out of 25 subjects were included. We lost 5 subjects due to lack of post-treatment specimen because of complete or close to complete eradication of their primary tumors, and we lost 3 other subjects due to the absence of consent for tissue staining. TILs were quantified and summarized for the 17 subjects (Figure 4A). H and E images representative of the scoring approach are shown (Supplementary Figure 1). The treatment schedule significantly affect percentage of stromal TILs (Figure 4A, *P* = 0.0325). Pair-wise comparisons of schedule C, median change of +5, with other schedules one-by-one shows a significant difference only with schedule D, median change of −10 (*P* = 0.0444). The change in TIL percentages after treatment in schedules C, median change of +5, showed a tendency towards significance when compared with other schedules combined, with median change of -2 (Figure 4B, *P* = 0.0819). More tissue slides were prepared and stained to detect predominant immune cell type among TILs. We did not observe NK (CD56+)-cell infiltration among the 17 subjects evaluated; however, the majority of TILs were CD3+ lymphocytes (Figure 4C). A similar pattern to TILs was observed when we examined the change in quantity of CD3+ T-cells (*P* = 0.0305, Figure 4C). Schedule C showed a positive change in in infiltrating CD3+ cells in residual lesions after treatment, median +4.5, that was significantly different than the median change in subjects treated in other schedules combined, median -2 (Figure 4D). We observed statistically significant change in the quantity of TILs and CD3+ cells after treatment affected by treatment schedule, showing a potential role for the peptide immunization. The data suggest that treatment schedule C may also positively affect infiltration of lymphocytes.

### Tumor response to combination therapy

**Figure 5:**
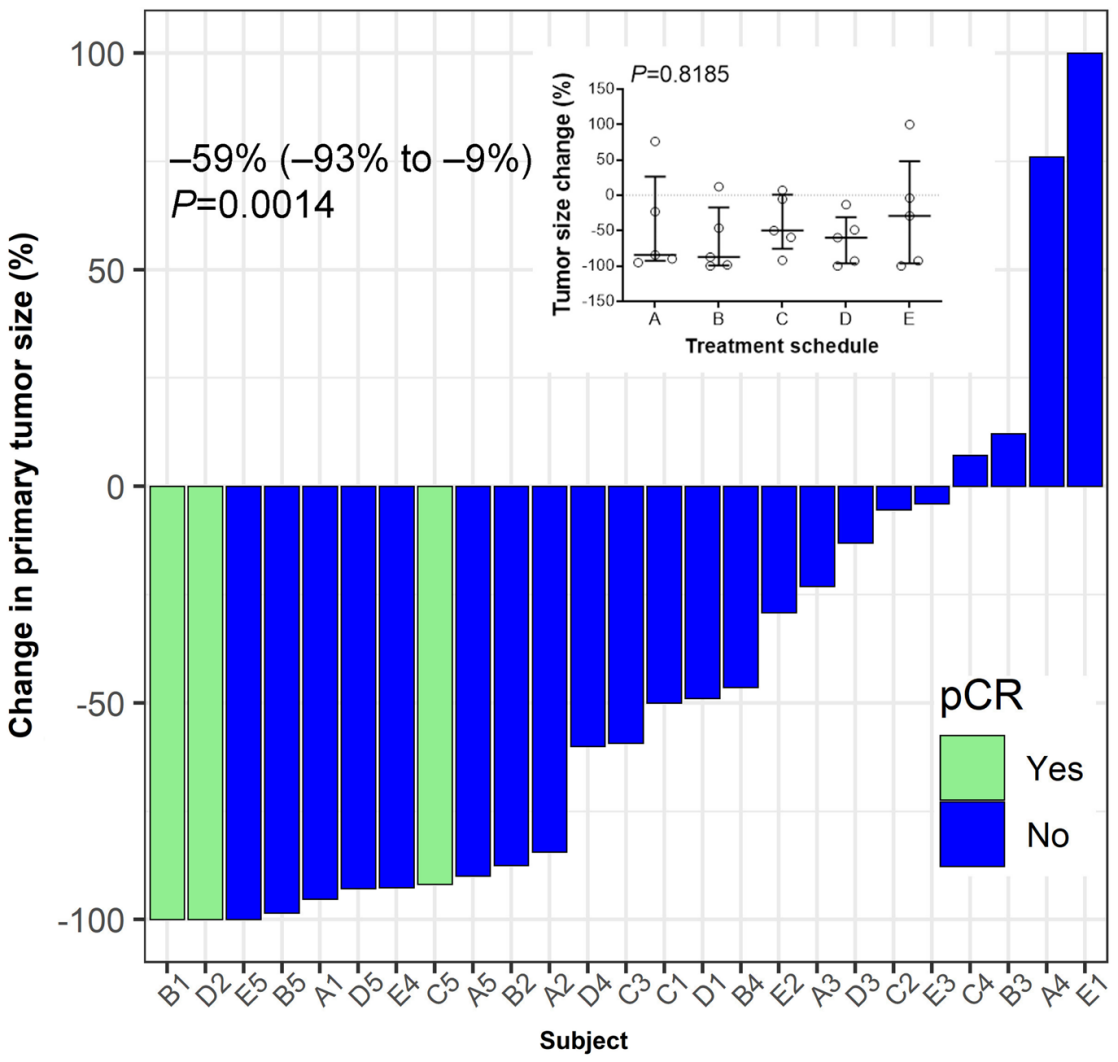
Tumor response to treatment. Hormone-receptor positive breast cancer patients were vaccinated for three consecutive weeks after enrolment in a neo-adjuvant setting. Pathological complete response (pCR) was defined as ypT0/Tis ypN0. Median (IQR, interquartile range) value of the percent change in the post-treatment tumor size from baseline at the time of surgery is shown. Wilcoxon’s signed-rank test was used to examine the statistical significance of tumor size reduction (*P* = 0.0014). Inset summarizes percent tumor-size change based on treatment schedule. No significant differences between schedules are observed (*P* = 0.8185). Bars indicate median and interquartile range.

To explore the potential impact of combination therapy on the tumor, pCR status and change in tumor size were investigated as clinical outcomes. We observed that combination therapy across the 5 cohorts resulted in a significant reduction in the primary size of the tumor (Figure 5). Three patients, one from each treatment schedule B, C, and D reached pCR. No statistically significant differences in tumor size change was observed comparing the 5 treatment schedules (Figure 5 inset).

## DISCUSSION

The main objective of our study was to determine an appropriate schedule to be used for adding the P10s-PADRE vaccine to cancer chemotherapy in the neoadjuvant setting considering the ability of the vaccine to elicit adequate antibody response. Three weekly immunizations with P10s-PADRE vaccine, added to NAC, were enough to generate antibodies in individuals treated in 5 different combination schedules. However, in one schedule, Schedule C, where the third immunization was administered a week before the first dose of chemotherapy, four out of 5 subjects responded, showing higher average fold increase in antibody titer. Therefore, the data indicate that the vaccine in combination with NAC is capable of generating antibody response early-on when administered immediately before standard-of-care chemotherapy. Establishing a high antibody titer quickly during patient treatment seems critical for combination with NAC. The follow-up data indicate that more subjects start to respond later into the cycles of NAC, emphasizing the immunological efficacy of the vaccine in combination with chemotherapy.

The majority of AEs were of grades 1 and 2, and all subjects fully completed their treatment. The safety data indicate that the combination therapy is feasible and well-tolerated.

When we analyzed CD3^–^/CD56^+^ cells among lymphocytes isolated from peripheral blood of the entire cohort, we found that the intensity of the CD16 expression was decreased after treatment. Analysis by schedule further revealed that treatment schedule affected CD16 expression, as subjects treated in schedule C, contrary to subjects in other schedules, showed an increase in the expression of CD16 on NK cells. The CD16 upregulation on NK cells in subjects enrolled in schedule C appears specific. CD16 can be a potent signal in elimination of antibody-coated tumor cells through ADCC, relevance of which should be investigated in future studies.

Contrary to the CD16 expression levels, NKp46 and CD94 receptors showed an overall increase in expression levels in the entire cohort. NKp46 is also a major receptor mediating cytotoxicity in fresh peripheral-blood NK cells [21] and is involved in controlling metastasis in a melanoma animal model [22]. It plays a role in the cytotoxic ability of these cells that is independent of antibodies [21] as signaling through NKp46 leads to secretion of IFN-γ and TNF-α [23] as well as other inflammatory cytokines that can directly affect tumor cells or ongoing adaptive immune responses [24]. No significant differences was observed between schedules regarding this marker suggesting a general response to the combination therapy. CD94 is another receptor family conserved in both mice and humans that can conjugate to most NKG2 family members [25]. CD94 can play both activation and inhibitory roles through conjugation with NKG2C or NKG2A [26]. Subjects in schedule C, show lower relative response to therapy in terms of CD94 expression. In our Phase I study performed on stage IV breast cancer patients [15], we have observed upregulation of NKp46 on NK cells upon immunization [16]. A role for the above markers in the efficacy of the combination therapy and their dependency on vaccination needs to be further investigated in future studies.

We observed higher IFN-γ in subjects treated in schedule B; the subjects that showed a significant reduction of CD16 expression. Others have shown that CD16 downregulation was associated with increased production of IFN-γ [27]. However, we did not observe differences between schedule C and other schedules regarding the increase in IFN-γ levels. The data suggest that the increase in IFN-γ, similar to NKp46, could be more of a consequence of the combination therapy. The activation of NK cells and an increase in IFN-γ could be a part of immune response to the NAC alone that is strongly associated with pre-chemo immune features [9] that could be influenced by vaccination. These immune features have significant implication for cancer immunotherapy, as both NK cells and IFN-γ release are considered major components of anti-cancer immune responses [28, 29]. The increase in IL-10, IL-6 and TNF-α though statistically significant, were small and not affected by treatment schedule.

Finally, we demonstrated that treatment schedules have different effect on the numbers of lymphocytes in the tumor microenvironment with schedule C showing higher increase in TILs in residual tumors after treatment. Promoting TILs may positively affect therapeutic modalities, improving long-term survival outcomes [10, 20]. The presence of TILs in residual disease after NAC was associated with better metastasis-free and overall survival [11]. Contrary to our expectation, we did not observe infiltration of NK cells. Instead, the data suggest an increase in stromal CD3+ T cells in residual tumors after treatment. The attraction of T cells into the tumor environment is consistent with our preclinical data showing T-cell-dependent DTH response and a role for T cells in tumor shrinkage using P10-KLH vaccine [30]. Whether more TILs can contribute to better survival outcomes needs to be addressed in future studies. Lack of enough tissues prohibited identification of T-cell subsets that is a limitation of our study.

## MATERIALS AND METHODS

### Study design

We conducted a Phase Ib clinical trial to assess safety, tolerability and feasibility of adding the CMP vaccine, P10s-PADRE [14, 15], to a standard-of-care chemotherapy in breast cancer subjects. This trial was approved by the Institutional Review Board (IRB) of the University of Arkansas for Medical Sciences (UAMS), and was registered with the NIH clinical-trials registry at http://clinicaltrials.gov (NCT02229084). Women 18 years of age or older, of all races, with clinical stage I, II or III ER+/HER2− breast cancer were eligible, and subjects were enrolled after providing written informed consent. Eligibility criteria are described in Supplementary Table 1. The patients were recruited from the Breast Cancer Clinic at the Winthrop P. Rockefeller Cancer Institute at the UAMS campus, and from the Oncology Clinic of the Highlands Oncology Group in Northwest Arkansas.

### Vaccine preparation, immunization and treatment schedule

P10s-PADRE was synthesized by AmbioPharm, Inc., (North Augusta, SC, USA) and administered with the adjuvant MONTANIDE™ ISA 51 VG STERILE (SEPPIC, Inc., Fairfield, NJ, USA) as described before [15]. For this study, we considered five different vaccination schedules (5 subjects per schedule), named A, B, C, D, and E, to evaluate the feasibility of eliciting an immune response when the vaccine was administered with NAC (Supplementary Table 2). The patients were immunized by administration of three weekly injections of P10s-PADRE vaccine at a dose of 500 μg. Patients were administered doxorubicin (60 mg/m^2^) and cyclophosphamide (600 mg/m^2^) every three weeks for four cycles followed by docetaxel (75 mg/m^2^) every three weeks for four cycles. The timing of immunizations relative to chemotherapy and blood draws for each schedule is shown in Supplementary Table 2.

### Endpoint variables

Safety and feasibility were the primary endpoints; assessing them was the primary objective. The immunological endpoints were anti-P10s antibody titers (primary) and NK cell activation (secondary). Tumor size was measured before treatment and at pathological examination after surgery, and change in the size of the primary tumor was calculated. Pathological examination and tumor measurements were performed blindly. Pathologic complete response (pCR) was defined as the absence of residual invasive cancer on histopathologic evaluation of paraffin-embedded tissue sections stained with hematoxylin and eosin of the complete resected breast specimen and all sampled regional lymph nodes following completion of the therapy (ypT0/Tis ypN0).

### Adverse event monitoring

Adverse events were assessed using the NCI Common Terminology Criteria for Adverse Events (CTCAE), Version 4.0.

### Serum collection and ELISA

Pre- and post-immunization serum samples were collected from each subject and anti-P10s-MAP IgG levels were measured via ELISA as described [14, 15]. The antibody titers were determined by measuring reactivity of 2-fold serial dilutions of both pre- and post-immune serum, and the fold change in anti-P10s-MAP IgG titer was determined in post-immune serum compared to baseline pre-immune serum.

### Cytokine measurement

The Meso Scale Discovery^®^ (MSD^®^) multi-spot V-PLEX^®^ assay system (Meso Scale diagnostics LLC, Rockville, MD, USA) was used to determine the levels of multiple cytokines in the serum samples collected according to manufacturer’s instructions. The Proinflammatory Panel 1 (human) kit, which tests for IFN-γ, IL-1β, IL-2, IL-4, IL-6, IL-8, IL-10, IL-12p70, IL-13, and TNF-α, was used for cytokine measurements. The V-PLEX^®^ assay plate was analyzed using the MSD Electrochemiluminescence charge-coupled device and QuickPlex SQ 120 imager and the results were reported using the Discovery Workbench 4.0 software.

### Isolation of peripheral blood mononuclear cells (PBMCs)

PBMCs were isolated from freshly collected blood using Histopaque-1077 (Sigma). Blood was carefully layered onto the Histopaque-1077 at a ratio of 1:1 and centrifuged at room temperature for 30 minutes. Following centrifugation, the opaque interface containing mononuclear cells was collected. The cells were washed three times with PBS. Fresh cells were used for staining in flow cytometry.

### Flow cytometry

Phenotypic studies were performed on isolated PBMCs by three-color flow cytometry using a BD LSRFortessa cytometer (BD Biosciences, San Jose, CA, USA), and analyzed using Flowjo^®^ software (Flowjo LLC, Ashland, Oregon). Lymphocytes were gated and CD3-negative CD56-positive lymphocytes were selected for further detection of NK cell receptor expression. The expression of CD16 (FCGR3, Fc fragment of IgG receptor III), CD69, NKp46 and CD94 (KLRD1, killer cell lectin-like receptor D1) on CD3-negative/CD56-positive cells was determined. The expression of CD69 on CD3+ and CD3+/CD56+ cells was also determined. All mAbs used for flow cytometry were purchased from BD Bioscienses (Becton, Dickinson and CompaFranklin lakes, NJ, USA).

### Tumor-infiltrating lymphocytes (TILs) and immunohistochemistry

TILs were assessed using H and E slides in FFPE sections obtained through core needle biopsies prior to therapy and through surgical specimens after treatment at surgery. Separate individual slides were stained with anti-CD56 (123C3.D5 + 123A8, Abcam plc, Cambridge, MA), anti-CD3 (SP7, Abcam plc), anti-CD8 (SP16, Abcam plc), anti-FoxP3 (1054C, R and D systems), and anti-CD68 (KP1, Abcam plc) antibodies. All slides were prepared and stained by the UAMS pathology core. The percentage of TILs was determined according to the method proposed by the International Immuno-Oncology Working Group [31, 32]. The same strategy was used to score various lymphocytes including CD56- and CD3-positive TILs. Slides were independently evaluated by two pathologists (SPO and GRP). Images were analyzed using a Nikon Eclipse Ni microscope at 20X magnification. Photomicrographs were taken with a Nikon DS-Fi3 microscope camera using the NIS-Elements D software from Nikon.

### Statistical assessments

All immune assays and evaluations of clinical endpoint were conducted blindly. Changes in immune indicators including antibody production, cytokine secretion and expression of NK markers were calculated and log-transformed for statistical analyses. GraphPad Prism 9 (San Diego, CA, USA) was used to conduct Wilcoxon signed-rank test, one-sample *t*-tests, and Kruskal-Wallis tests with post-hoc analyses using Dunn’s multiple-comparison procedure. Fold increases in anti-P10s peptide titer were analyzed using the 1-sided one-sample *t*-test at α = 0.05 to compare mean fold increases against the null-hypothesis value of a 4-fold increase. All other statistical tests were 2-sided at α = 0.05.

### Ethics approval and consent to participate

The clinical trial and all procedure were performed after approval by the UAMS IRB (reference number 202556) and in accordance with an assurance filed with and approved by the Department of Health and Human Services. The study is registered with the NIH clinical-trials registry at http://clinicaltrials.gov (NCT02229084). Informed consent was obtained from all individuals included in the study.

### Consent for publication

Subjects were consented for dissemination and communication of de-identified data generated in the study.

### Data availability

All data relevant to the study are included in the article or provided as supplementary materials.

## CONCLUSIONS

This Phase Ib clinical trial of the P10s-PADRE vaccine shows that immunization in combination with a standard-of-care NAC is feasible and well-tolerated. Combination therapy induces antibody response, stimulates activation of NK cells, and is associated with infiltration of T cells in tumor microenvironment. Randomized phase II trials focusing on treatment schedule C are needed to validate current findings and evaluate clinical efficacy.

## SUPPLEMENTARY MATERIALS



## Abbreviations

ADCC: antibody-dependent cell-mediated cytotoxicity
CMP: carbohydrate mimicking peptide
FCGR3: Fc fragment of IgG receptor III
HR+: hormone receptor-positive
HER2-: human Epidermal Receptor 2 negative
IgG: immunoglobulin G
IRB: institutional review board
KLRD1: killer cell lectin like receptor D1
NAC: neoadjuvant chemotherapy
NCAM1: neural cell adhesion molecule 1
NCR1: natural cytotoxicity triggering receptor 1
NK cells: natural killer cells
pCR: pathological complete response
PADRE: pan DR T helper epitope
SC: subcutaneous
TILs: tumor-infiltrating lymphocytes
UAMS: University of Arkansas for Medical Sciences
