# Supplementary material for: P10s-PADRE vaccine combined with neoadjuvant chemotherapy in ER-positive breast cancer patients induces humoral and cellular immune responses
Source: Oncotarget. 2021 Oct 26;12(22):2252–65. doi: 10.18632/oncotarget.28083 (PMC8555684; doi:10.18632/oncotarget.28083)
Supplement: Supplementary file 1 [file oncotarget-12-2252-s001.pdf]

P10s-PADRE vaccine combined with neoadjuvant chemotherapy in ER-positive breast cancer patients induces humoral and cellular immune responses

SUPPLEMENTARY MATERIALS

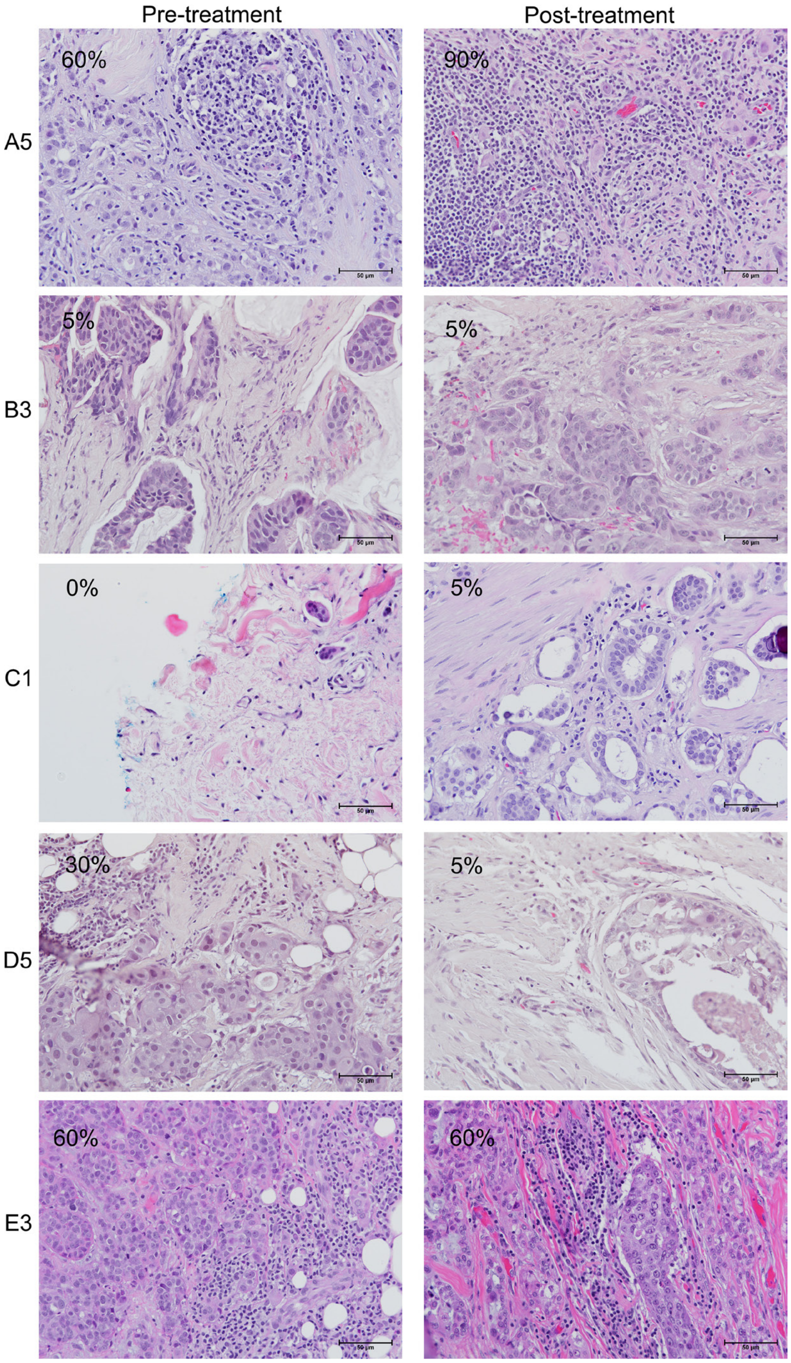

Supplementary Figure 1: TILs assessment. Images of representative tumor specimens sampled pre- and post-immunization from each schedule are illustrated. Percent TILs assessed for each specimen is shown.

Supplementary Table 1: Inclusion and exclusion criteria

Inclusion criteria

- Females of all races with clinical stage I, II, or III ER-positive, HER2-negative breast cancer who will undergo standard neoadjuvant treatment.
- Age 18 years and older.
- ECOG Performance Status 0 or 1.
- White blood cell (WBC) count ≥ 3,000/mm^3 within 3 weeks prior to registration.
- Platelet count ≥ 100,000/mm^3 within 3 weeks prior to registration.
- Bilirubin ≤ 2 x institutional upper limit (IUL) of normal obtained within 3 weeks prior to registration.
- Serum glutamic-oxaloacetic transaminase (SGOT) or aspartate aminotransferase test (AST) ≤ 2 x IUL of normal obtained within 3 weeks prior to registration.
- Serum glutamic-pyruvic transaminase (SGPT) or alanine aminotransferase test (ALT) ≤ 2 x IUL of normal obtained within 3 weeks prior to registration.
- Serum creatinine ≤ 1.8 mg/dL obtained within 3 weeks prior to registration.
- Must sign an informed consent document approved by the UAMS IRB.

Exclusion criteria

- Active infection requiring treatment with antibiotics.
- Existing diagnosis or history of organic brain syndrome that might preclude participation in the full protocol.
- Existing diagnosis or history of significant impairment of basal cognitive function that might preclude participation in the full protocol.
- Other current malignancies. Subjects with prior history at any time of any in situ cancer, including lobular carcinoma of the breast in situ, cervical cancer in situ, atypical melanocytic hyperplasia or Clark I melanoma in situ or basal or squamous skin cancer are eligible, provided they are disease-free at the time of registration. Subjects with other malignancies are eligible if they have been continuously disease free for ≥ 5 years prior to the time of registration.
- Active autoimmune disorders or conditions of immunosuppression; Existing diagnosis or history of autoimmune disorders or conditions of immunosuppression that have been in remission for less than 6 months
- Treatment with corticosteroids, including oral steroids (i.e. prednisone, dexamethasone [except when used as an antiemetic in standard therapy]), continuous use of topical steroid creams or ointments or any steroid-containing inhalers. Subjects who discontinue the use of these classes of medication for at least 6 weeks prior to registration are eligible if, in the judgment of the treating physician, the subject is not likely to require these classes of drugs during the treatment period. Replacement doses of steroids for subjects with adrenal insufficiency are allowed.
- Pregnancy or breastfeeding (due to the unknown effects of peptide mimotope vaccines on a fetus or infant). Women of childbearing potential must have a negative urine pregnancy test within 72 hours prior to starting week 1 and must be counseled to use an accepted and effective method of contraception (including abstinence) while on treatment and for a period of 18 months after completing or discontinuing treatment. Accepted methods of contraception include tubal ligation, oral contraceptives, barrier methods, IUDs, and abstinence.
- Any other significant medical or psychiatric conditions, which, in the opinion of the enrolling investigator, may interfere with consent or compliance of the treatment regimen.
- Enrollment in any other clinical trial using investigational drug products or devices.

Supplementary Table 2: Immunization timing and blood draws relative to drugs used in various schedules (cohorts A, B, C, D, E)

Procedures	Cohorts				
	A	B	C	D	E
**Vaccination with Mimotope P10s-PADRE/MONTANIDE™ ISA 51 VG**	Week 1, 2 and 3	Week 2, 3 and 4	Week 1, 2 and 3	Week 1, 2 and 3	Week 1, 2 and 3
**Cyclophosphamide**	Week 1, 4, 7 and 10	Week 1, 4, 7 and 10	Week 4, 7, 10 and 13	Week 2, 5, 8 and 11	Week 3, 6, 9 and 12
**Doxorubicin**	Week 1, 4, 7 and 10	Week 1, 4, 7 and 10	Week 4, 7, 10 and 13	Week 2, 5, 8 and 11	Week 3, 6, 9 and 12
**Docetaxel**	Week 13, 16, 19, and 22	Week 13, 16, 19 and 22	Week 16, 19, 22 and 25	Week 14, 17, 20 and 23	Week 15, 18, 21 and 24
**Blood draw**	Week 1, 4, **7**, 10, 13, 22, 46 and 70	Week 1, 7, **10**, 13, 16, 22, 46 and 70	Week 1, 7, **10**, 13, 16, 25, 49 and 73	Week 1, 5, **8**, 11, 14, 23, 47 and 71	Week 1, 6, **9**, 12, 15, 24, 48 and 72

Surgery was performed 4–8 weeks after the last taxol injection. Blood samples collected 4–6 weeks after third immunization (Bold) were used for flow cytometry and cytokines.
